# Split NanoLuc technology allows quantitation of interactions between PII protein and its receptors with unprecedented sensitivity and reveals transient interactions

**DOI:** 10.1038/s41598-021-91856-2

**Published:** 2021-06-15

**Authors:** Rokhsareh Rozbeh, Karl Forchhammer

**Affiliations:** Interfaculty Institute of Microbiology and Infection Medicine, University of Tübingen, Auf der Morgenstelle 28, 72076 Tübingen, Germany

**Keywords:** Biological techniques, Biotechnology, Microbiology, Molecular biology

## Abstract

PII proteins constitute a widespread signal transduction superfamily in the prokaryotic world. The canonical PII signal proteins sense metabolic state of the cells by binding the metabolite molecules ATP, ADP and 2-oxoglutarate. Depending on bound effector molecule, PII proteins interact with and modulate the activity of multiple target proteins. To investigate the complexity of interactions of PII with target proteins, analytical methods that do not disrupt the native cellular context are required. To this purpose, split luciferase proteins have been used to develop a novel complementation reporter called NanoLuc Binary Technology (NanoBiT). The luciferase NanoLuc is divided in two subunits: a 18 kDa polypeptide termed “Large BiT” and a 1.3 kDa peptide termed “Small BiT”, which only weakly associate. When fused to proteins of interest, they reconstitute an active luciferase when the proteins of interest interact. Therefore, we set out to develop a new NanoBiT sensor based on the interaction of PII protein from *Synechocystis* sp. PCC6803 with PII-interacting protein X (PipX) and N-acetyl-L-glutamate kinase (NAGK). The novel NanoBiT sensor showed unprecedented sensitivity, which made it possible to detect even weak and transient interactions between PII variants and their interacting partners, thereby shedding new light in PII signalling processes.

## Introduction

PII signalling proteins are ubiquitous in nature, in particular in Prokaryotes and plastids of Archaeplastida^1^. Their general task is to sense the metabolic state of the cells by binding in an interactive manner the metabolite status reporter molecules ATP and ADP as well as 2-oxoglutarate^2^. Effector molecule binding results in different conformational states of PII proteins, in particular, of their large flexible T-loops acting as versatile protein–protein interaction modules. Depending on the metabolite binding status, and thus, the PII-conformation, various PII receptors read out the metabolic information processed by PII. This is achieved through transient interactions with PII, which alter the activities of the PII-receptor proteins. Although initially thought to be confined to the regulation of nitrogen metabolism, research in the past years has unveiled a plethora of PII regulated proteins^2,3^.

The focus of our work has been on the PII regulated processes in cyanobacteria. These are oxygenic photosynthetic, autotrophic bacteria playing important roles in global carbon- and nitrogen cycles. The first PII interaction partners, the N-acetyl-L-glutamate kinase (NAGK), a key enzyme of arginine synthesis, and PipX, a transcriptional co-activator of the global nitrogen control transcription factor NtcA, were identified by yeast-two hybrid screening^4,5^. Detailed mechanistic insight in the interaction of PII with these receptors has been obtained by solving the X-ray crystallographic structures of the proteins in complex^6,7^, and characterizing the PII interactions mainly through Surface-Plasmon-Resonance analysis^8–11^. Even more details have been obtained by FRET-based quantitative analysis by fusing PII and NAGK or PipX to fluorescent proteins^12–14^.

More recently, mainly through PII-co-purification approaches, novel interaction partners of PII proteins have been identified in cyanobacteria, such as the biotin carboxyl carrier protein (BCCP)-subunit of the Acetyl-CoA carboxylase^15^, an ensemble of nitrogen uptake systems (the nitrate transport system NRT, urea-transport system URT and ammonium transport protein AMT1)^16^, the Phosphoenolpyruvate carboxylase (PepC)^17^ and two regulatory peptides, termed PirC and PirA, the former regulating carbon flow through interaction with the Phosphoglycerate mutase (PGAM), and the second, the arginine synthesis pathway^18,19^. However, for all the latter interaction partners, structural information is not yet available. Of the recently identified PII-receptors*, *in vitro protein interaction analysis has been performed for BCCP, PEPC, PirC and PirA. A striking feature of PII–PII-receptor interactions is in the ability of the same effector molecule to differentially affect the interaction of PII with different receptors. For example, PII binds NAGK in the absence of effector molecules. Binding of ADP to PII increases complex dissociation^10^, and ATP in combination with 2-OG completely prevents complex formation^20^. By contrast, ADP enhances the stability of the PII–PipX complex, whereas ATP together with 2-OG again prevents complex formation. When small amounts of ADP were added to Mg-ATP/2-OG pre-loaded PII, PipX was again able to bind to PII, whereas small amounts of ADP did not affect PII-NAGK interactions in presence of ATP^10^. Structural analysis provided a mechanistic explanation: Formation of the PII-PipX complex stabilizes the conformation of PII that preferentially binds ADP. By contrast, PII in the NAGK complex adopts a conformation that preferentially binds ATP but is incompatible with 2-OG binding^11^. As a consequence, interaction between PII and PipX is highly sensitive to fluctuations in the ATP/ADP ratio whereas PII -NAGK complex responds mainly to changes to the 2-oxoglutarate levels, as long as sufficient ATP is present. This allows PipX to sense the energy status via PII interaction whereas at the same time, NAGK can read out the level of 2-oxoglutarate (a status reporter of the carbon/nitrogen balance), provided that PII is present in excess over the receptors, which appears to be the case^21^.

A limitation of the interaction analyses using Surface-Plasmon-Resonance (SPR) or Biolayer interferometry (BLI) comes from the fact that one interaction partner has to be immobilized on a sensor-surface, restricting access and mobility of the proteins. Indeed, fixing PII via a C-terminal Strep-tag to the surface of a Biacore sensor disturbed the response of the PII-PipX complex to ATP and 2-oxoglutarate^10^. Likewise, the use of FRET based analytics requires the fusion of large fluorescence proteins to both interaction partners and is highly sensitive to background fluorescence^22^. An alternative is the use of protein fragment complementation assays (PCA). PCA is a powerful approach to determine protein–protein interactions and has been applied in various variants^23^. A very attractive PCA version that was recently developed to determine PII protein interactions in solution is the split NanoLuc system. NanoLuc is an engineered luciferase enzyme from the deep ocean shrimp with exceptionally bright bioluminescence^24^. A variant of the enzyme was further engineered as protein fragment complementation reporter^25^, now commercially available as NanoLuc Binary Technology (NanoBiT)^26^. The small 11 amino acids fragment (termed Small BiT, SmBiT) was constructed in a way that it has low intrinsic affinity (190 µM) towards the large complementation fragment (Large BiT, LgBiT). When LgBiT and SmBiT fragments are fused to candidate interacting proteins, luminescence is only restored when the candidate proteins interact with each other.

Recently, the NanoBiT technology was used to quantitatively analyse several protein–protein interactions in mammalian cells. It was used to verify the interaction between SME1 β-lactamase and a set of inhibitor binding proteins in vivo with the optimal performance when LgBiT was attached to the C-terminus of SME1 and SmBiT was attached to the C-terminus of inhibitor binding proteins^25^. The system was also used to accurately characterize the interaction between bacterial transcription factors NusB and NusE, was well as the interaction between RNA polymerase and σA from *Bacillus subtilis*^27^. Here, we employed NanoBiT technology to analyse the interactions of PII with PipX and NAGK, and show that this method provides superior quantitative results with exceptionally high sensitivity in comparison to SRP or BLI. These analyses also provide additional insights into the sophisticated network of PII interactions.

## Results

### Development of the NanoBiT sensor

**Figure 1:**
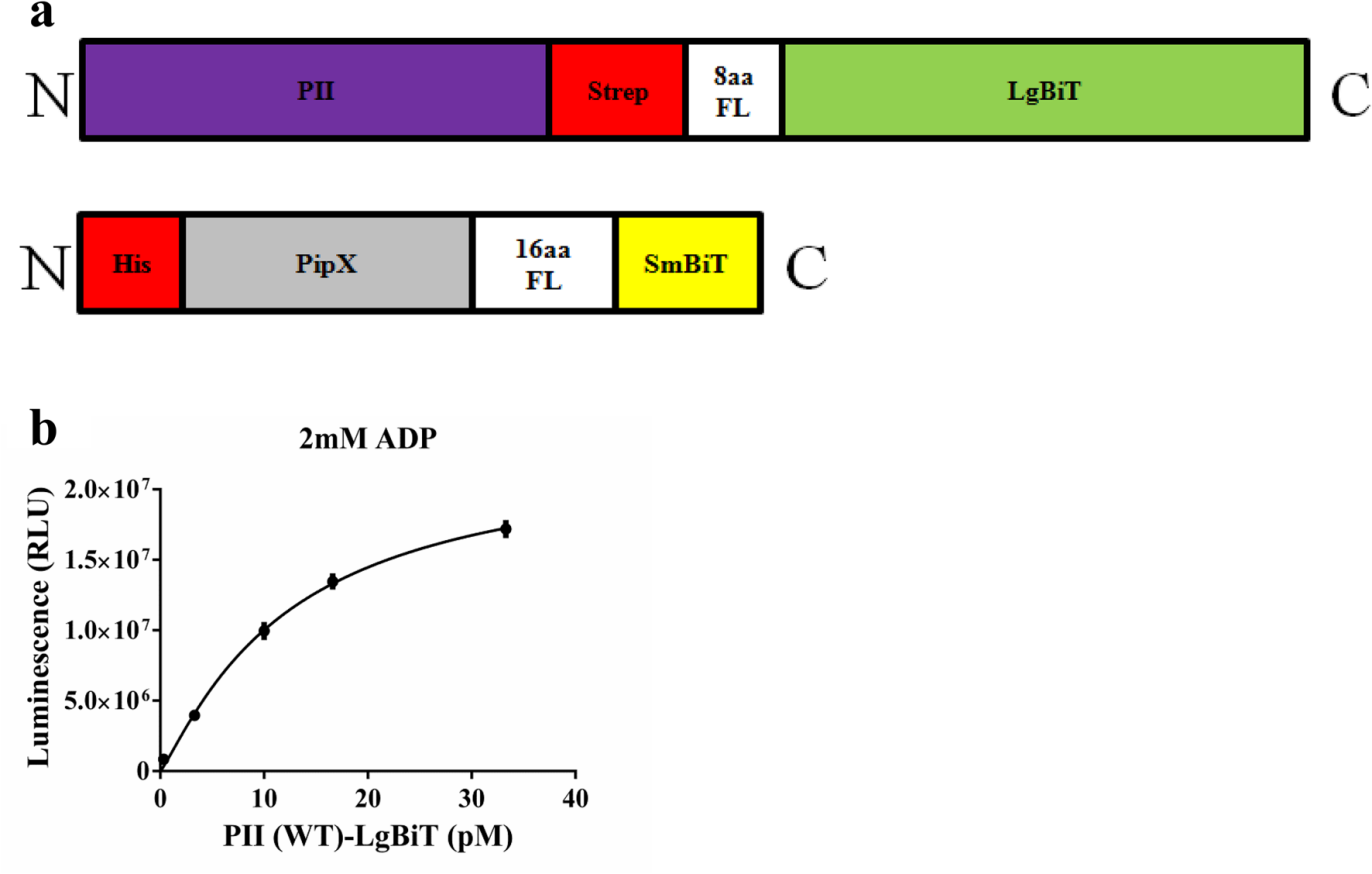
Fusion protein constructs and NanoBiT responce*.* (**a**) Schematic representation of PII-LgBiT and PipX-SmBiT fusion proteins. Flexible linker with 16 amino acids (16aa) in construct fused to SmBiT. (**b**) Titration of PII-LgBiT (0.33–33 pM) with constant amount of PipX-SmBiT (10 nM) in the presence of 2 mM ADP. Graph represents mean ± SD of three independent experiments.

Initially we constructed hybrid PII fusion proteins that contained LgBiT fragment at the C-terminus and SmBiT fragments were fused to the tip of the T-loop with three different linkers (24aa linker, 16aa linker and 8aa linker) in a similar manner than previously reported for hybrid PII-FRET constructs^22^. Of these constructs, only the 16 aa linker construct showed a response to the addition of PII effector molecules, however with only a response of 30% signal difference (data not shown). Therefore, we decided to separate the NanoBiT fragments in two distinct polypeptites: on PII and a PII-receptor protein, using the 16 aa linker to fuse the SmBiT fragment, which appeared most suitable from the initial experiments. The LgBiT fragment was fused at the C-terminally located Strep-tag purification linker of PII, which was already successfully used as linker for PII-VENUS FRET constructs^12^. The SmBiT fragment was fused at the C-terminus of PipX, with a 16 amino acids flexible linker in between (Fig. 1a). The gene for the PipX-SmBiT construct was cloned into expression vector pTEV5, containing an N-terminal His_6_ affinity tag. When expressed in *E.coli* the recombinant proteins formed inclusion bodies. These were dissolved in 8 M urea for His-tag affinity purification, and the purified proteins were subsequently re-folded. Purified, refolded PipX-SmBiT and PII-LgBiT alone showed low levels of background luminescence _~_ 1 × 10^−5^ RLU (relative light unit). This background was subtracted from the luminescence signals of the subsequent assays. In initial trials, PipX-SmBiT was used at a concentration of 10 nM and PII-LgBiT was titrated at increasing concentrations starting with 0.33 pM in a buffer containing 2 mM ADP, which are the conditions that were previously shown to be optimal for PII-PipX interaction^10^. After 15 min of incubation at 30 °C, the luminescence signal was recorded and plotted against the respective PII-LgBiT concentration. As shown in Fig. 1b, at low PII-LgBiT concentrations, a linear increase of luminesce up to 1 × 10^7^ RLU was detected (corresponding to 10 pM PII) whereas the curved flattened at higher concentration of PII-LgBiT. This indicates that the higher concentrations of NanoLuc are beyond the linear detection range of the assay. Therefore, in all subsequent assays the concentration of PII-LgBiT was limited to 10 pM.

### PII-PipX binding kinetics and influence of effector molecules

**Figure 2:**
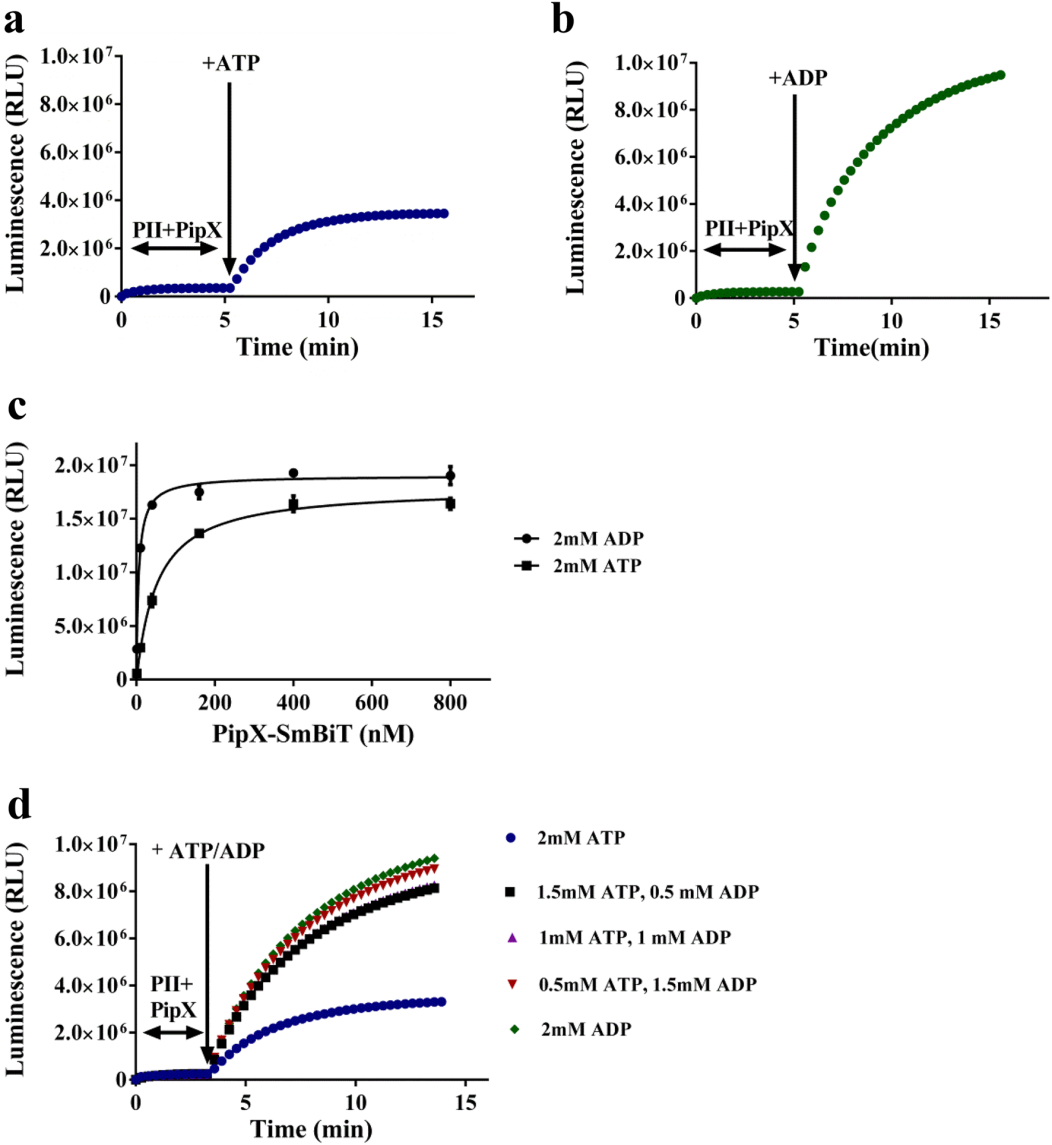
Effect of ATP or ADP on PII-LgBiT-PipX-SmBiT complex formation. (**a** and **b**) Time course of complex formation after adding 2 mM ATP (**a**) or 2 mM ADP (**b**). (**c**) Titration of PipX-SmBiT (0, 1, 10, 40, 160, 400, 800 nM) to PII-LgBiT (10 pM) in the presence of 2 mM ATP or 2 mM ADP. (**d**) Time course of complex formation PII-LgBiT (10 pM) and PipX-SmBiT (10 nM) in the presence of different ATP/ADP mixtures at a total ADP + ATP concentration of 2 mM. Graphs represent mean ± SD of three independent experiments.

The next step was to monitor the kinetics of PII-PipX complex formation in real-time by continuously measuring the luminescence in the presence of various PII effector molecules. Therefore, PII-LgBiT (concentration of 10 pM trimer) was first mixed with the indicated amounts of PipX-SmBiT and luminescence was measured for five minutes without effector molecules. As shown in Fig. 2a,b, this resulted only in a very low increase in luminescence. Subsequent addition of adenyl-nucleotides immediately stimulated the interaction as shown by the rise of luminescence signal (Fig. 2a,b). With 2 mM ATP, complex formation reached a plateau after about 10 min, indicating equilibrium between complex association and dissociation. In presence of ADP, the increase was much steeper, and 10 min after ADP addition, equilibrium was not yet reached, with a maximal signal for the ADP-promoted complex 2,5 times higher than that of the ATP-promoted complex.

To quantitatively determine the dissociation constant of PII-PipX interaction in presence of 2 mM ATP or ADP, increasing concentrations of PipX were titrated to a fixed concentration of PII-LgBiT (10 pM trimer). Then, the RLUs were plotted against the PipX-SmBiT concentrations (Fig. 2c). From these curves, K_D_ values were calculated assuming that in equilibrium the dissociation constant follows the Eq. (1):

$${\text{K}}_{{\text{D}}} = \left[ {{\text{PII}} - {\text{LgBiT}}} \right]{ \times }\left[ {{\text{PipX}} - {\text{SmBiT}}} \right]/\left[ {{\text{PII}} - {\text{LgBiT}} * {\text{PipX}} - {\text{SmBiT}}} \right].$$

At the concentration of PipX-SmBiT at which half maximal luminescence is observed, 50% of PII should be in the PipX complex. This concentration should therefore correspond to the K_D_ of the complex (provided that PipX is in large excess over PII so that the free PipX concentrations approximately equal the total PipX concentration). We estimated the half-maximal RLU value by hyperbolic fitting, resulting in a K_D_ of 5.7 ± 0.78 nM for the PII-PipX complex in presence of ADP and of 52.4 ± 0.92 nM in presence of ATP (Table 1).

**Table 1:** Dissociation constants (nM) of PII-LgBiT fusion variants with NAGK-SmBiT and PipX-SmBiT in presence of different metabolites.

Protein complex	Effector molecules condition and K_D_ (nM) of protein complex									
	2 mM ATP	2 mM ADP	No effector	2 mM ATP/ 2 mM Arg	2mMATP/ 2mMNAG	2 mM ATP/ 50 mM NAG	2 mM ATP/ 2 mM Arg/ 2 mM NAG	2mM ATP/2 mM Arg/50 mM NAG	2 mM ADP/ 2 mM NAG	2 mM ADP/ 50 mM NAG
PII (WT)-NAGK	8.8 ± 0.72	30.8 ± 0.12	12.9 ± 0.65	17.8 ± 0.72	4.7 ± 0.35	0.74 ± 0.081	16.5 ± 1.08	9.5 ± 0.20	14.9 ± 1.13	1.2 ± 0.053
PII(S49E)-NAGK	155 ± 0.37	95.3 ± 1.05	133 ± 0.62	432 ± 1.34	10.7 ± 0.91	3.39 ± 0.73	nm	nm	nm	nm
PII (I86N)-NAGK	7 ± 0.20	11.7 ± 0.14	12.9 ± 0.20	1.56 ± 0.48	4.1 ± 0.33	0.68 ± 0.031	nm	nm	nm	nm
PII (WT)- PipX	52 ± 0.92	5.7 ± 0.78	nd	nm	nm	nm	nm	nm	nm	nm

Data are the mean ± SD of triplicate measurements. The raw data was fitted using one site-specific binding with hill slope. *nd* not detectable; *nm*: not measured

The ATP to ADP ratio is an indicator of the cellular energy state. To determine its impact on PII-PipX interaction as revealed by NanoBiT analysis, the real-time luminescence increase was recorded at various ATP/ADP ratios, keeping the total ADP + ATP concentration constant at 2 mM. As shown in Fig. 2d, ADP is the dominant effector over ATP. Already 0.5 mM ADP in presence of 1.5 mM ATP (ATP/ADP ratio 3) is sufficient to increase luminescence signal up to 80%. The result reported in this study matches a previous similar experiment performed by SPR, which also showed that the interaction between PII and PipX is highly sensitive to fluctuations in the ATP/ADP ratios^11^. Strongly fluctuating ATP to ADP levels in Synechocystis cells have been reported in particular in response to light – dark shifts or different nutritional conditons^28–30^, Therefore, the response of PII-PipX interaction to differing ATP-ADP ratios seems physiologically relevant and matches the observations reported by Espinosa et al^29^.

**Figure 3:**
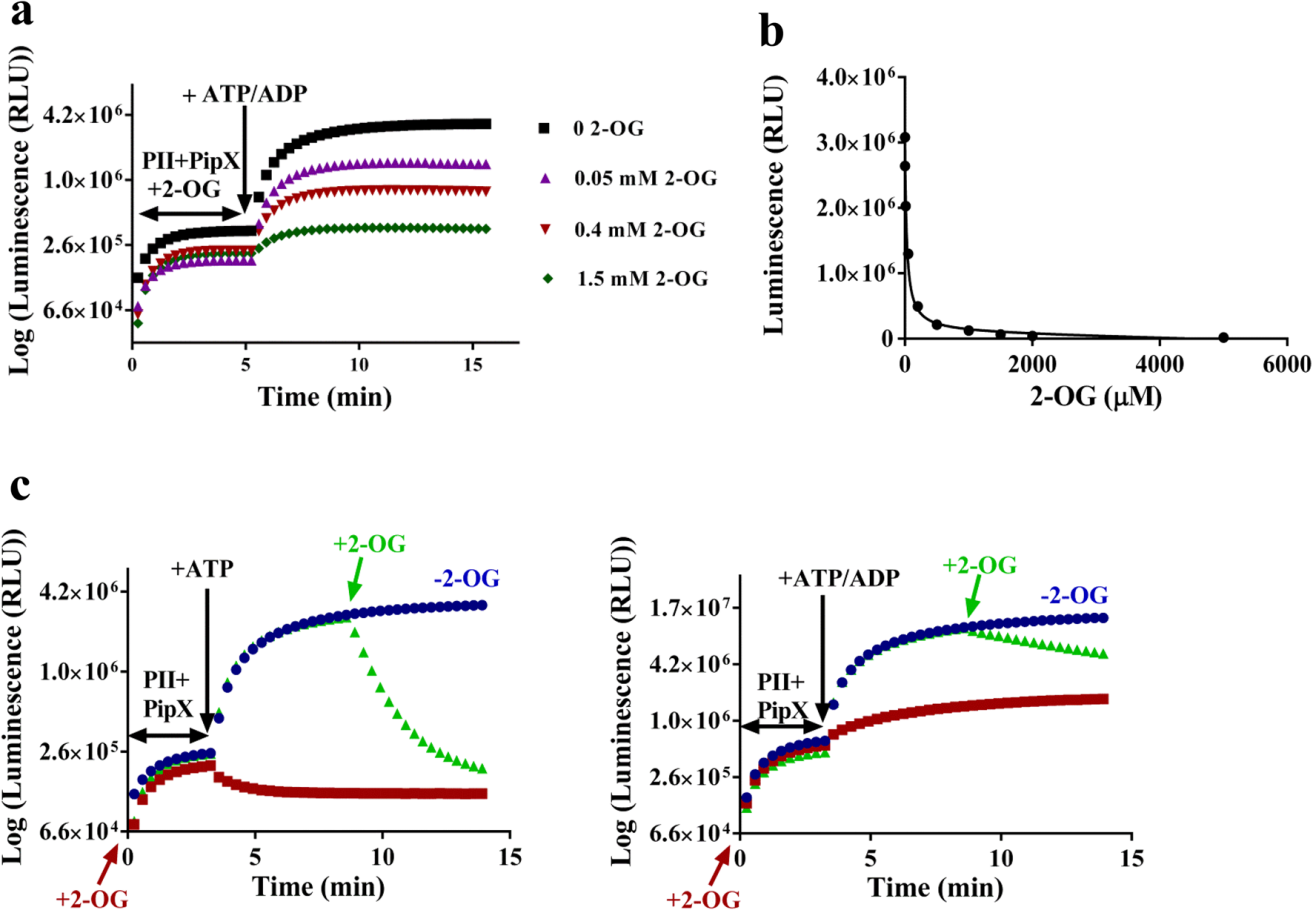
Effect of 2-OG on PII-LgBiT (10 pM) and PipX-SmBiT (10 nM) complex formation. (**a**) Interaction of PII-LgBiT and PipX-SmBiT in the presence of: none, 0, 0.05 mM, 0.4 mM and 1.5 mM 2-OG. (**b**) Quantitative analysis of 2-OG dependent inhibition of complex formation. (**c**) Time course of complex dissociation after addition of 2 mM 2-OG at indicated time point (green symbols). Control without 2-OG addition (blue symbols). First, PII-LgBiT and PipX-SmBiT were incubated without effectors (blue and green) or with only 2-OG (red symbols). After 4 min, either ATP (left) or 1 mM ATP and 1 mM ADP (right) were added, followed by the addition of 2-OG.

Next, we studied the effect of different 2-OG concentrations on PII-PipX complex formation (10 pM PII and 10 nM PipX) in the presence of 2 mM ATP. First, the mixtures of PII-LgBiT and PipX-SmBiT were incubated with the various 2-OG concentrations in the absence of ATP. After 5 min, 2 mM ATP was added and complexes formed until reaching saturation after 15 min (Fig. 3a). To quantify the effect, the maximal RLU values were plotted against the respective 2-OG concentrations (0, 0.002, 0.01, 0.05, 0.2, 0.5, 1, 1.5, 2, 5 mM) in the assay (Fig. 3b). In a similar way as reported by SPR, increasing 2-OG concentrations gradually prevented complex formation. Curve fitting resulted in an IC_50_ of 25 µM _(_corresponding to a 50% signal drop) for the inhibition by 2-OG (Fig. 3b). This value is in a similar range, albeit 40% lower than the average K_D_ of 2-OG binding to PII-Mg-ATP (39 µM)^10^, whereas the K_D_ of 2-OG binding to the first (highest affinity) binding site in trimeric PII is approx. 5 µM. This result indicates that trimeric PII with the highest affinity site occupied by 2-OG can still bind PipX via its free subunits, and only when fully occupied by 2-OG, PII is impaired in PipX binding.

To study the kinetics of 2-OG promoted PII-PipX complex dissociation with the NanoBiT technology, the luminescence signal dynamics was recorded in time-course experiments in which effector molecules were added successively (Fig. 3c). First, PII-LgBiT and PipX-SmBiT were pre-incubated without adenyl-nucleotides (green and blue symbols) or in a control experiment, in the presence of 2 mM 2-OG (red symbols). After four minutes, 2 mM ATP (left panel) or 1 mM ATP + 1 mM ADP (right panel) were added to allow complex formation. Five minutes later, 2 mM 2-OG was added to one sample each (green symbols) and the luminescence signal was recorded for further five minutes. In the presence of ATP alone, 2-OG completely dissociated the complex within two minutes to basal levels. After 45 s, about half of the complex was dissociated. A different response was observed in the presence of 1 mM ATP + 1 mM ADP, where dissociation was strongly mitigated. It required approximately 340 s to dissociate half of the complexes to basal level, 7–8-times longer than in the absence of ADP. The K_D_ of 2-OG binding to PII in presence of 1 mM ATP + 1 mM ADP was previously determined to be 180 µM, compared to about 39 µM in presence of 2 mM ATP ^10^. Therefore, the slower dissociation of the PII-PipX complex is in accord with the decreased affinity of PII for 2-OG in presence of 1 mM ATP/ADP.

### PII-NAGK binding kinetics and influence of effector molecules

**Figure 4:**
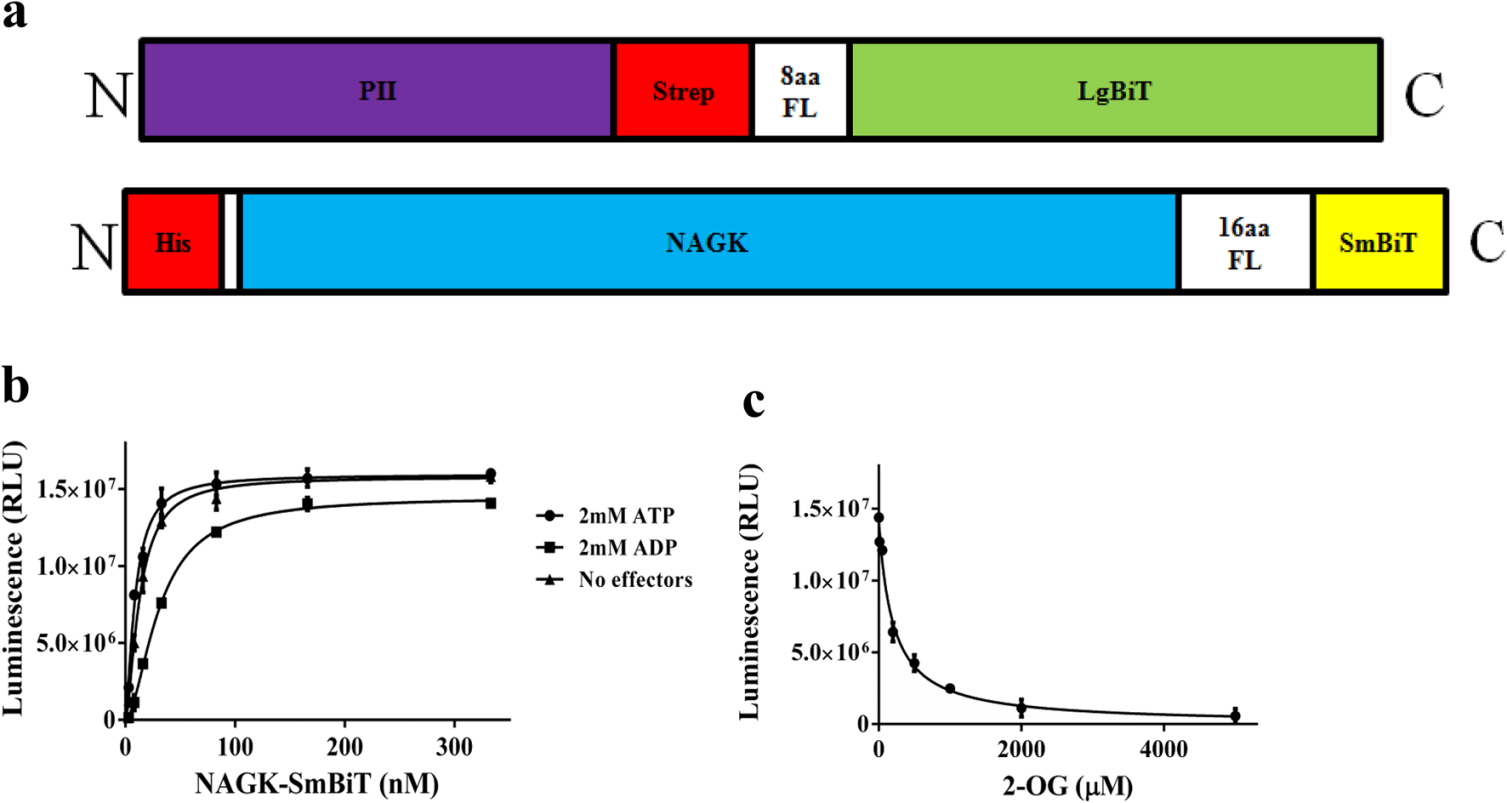
NanoBiT pair consisting of PII-LgBiT-NAGK-SmBiT. (**a**). Schematic representation of NanoBiT-sensor construct as indicated (**b**) Titration of NAGK-SmBiT in the range of 3–333 nM with 10 pM PII-LgBiT in the presence of 2 mM ATP or 2 mM ADP or in the absence of effector molecules. (**c**) Inhibitory effect of 2-OG (0, 0.01, 0.05, 0.2, 0.5, 1, 2, 5 mM) on PII-LgBiT (10 pM) and NAGK-SmBiT (27 nM) complex formation in the presence of 2 mM ATP. Graphs represent mean ± SD of three independent experiments.

To find out whether NanoBiT can also be applied to directly study PII interaction with other receptor N-acetyl-L-glutamate kinase (NAGK), the second well-characterized cyanobacterial PII-receptor, a PII-LgBiT-NAGK-SmBiT pair was developed. Therefore, the SmBiT fragment together with the 16 amino acids flexible linker was fused to the C-terminus of NAGK (Fig. 4a). As a first experiment, the purified NAGK-SmBiT fusion protein was titrated to 10 pM PII-LgBiT in the presence of 2 mM ATP, 2 mM ADP or in the absence of effector molecules (Fig. 4b). Efficient complex formation was detected between PII-LgBiT and NAGK-SmBiT in the absence of effector molecules and in presence of ATP. Complex formation also occurred in the presence of ADP, albeit with lower affinity (Fig. 4b). Hyperbolic fitting resulted in apparent K_D_ values of 12.9 ± 0.65 nM in the absence of effectors, 8.8 ± 0.72 nM in the presence of ATP and 30.8 ± 0.12 nM in the presence of ADP. In SPR experiments, only weak and transient PII-NAGK complexes could be detected in presence of ADP, which prevented quantitative determination^10^. This result demonstrates superior sensitivity of the NanoBiT over SPR for the detection and quantification of weak interactions.

To monitor the effect of 2-OG on PII-NAGK complex formation, 10 pM PII-LgBiT trimer was incubated with 160 nM NAGK-SmBiT (26.6 nM hexameric NAGK) in the presence of 2 mM ATP and different concentrations of 2-OG. Preliminary experiments showed that to achieve equilibrium, at least 30 min incubation time was necessary. Therefore, for quantification of 2-OG effects, the luminescence reagent was added 40 min after the start of PII-LgBiT-NAGK-SmBiT complex formation. Then, luminescence was recorded and plotted against the 2-OG concentrations (Fig. 4c). Similar to what observed for the PII–PipX complex, the inhibitory effect of 2-OG was clearly revealed by the NanoBiT assay. The IC_50_ for 2-OG, which led to 50% drop in luminescence, was determined to be 0.15 mM. This value correlated well with previous data obtained through different experimental approaches. Determination of PII-NAGK interaction by FRET assay revealed an IC_50_ for 2-OG of 0.1 mM^12^, and SPR and enzyme assays yielded an IC_50_ of 0.12mM^20^.

### Competition between PipX and NAGK for binding to PII

**Figure 5:**
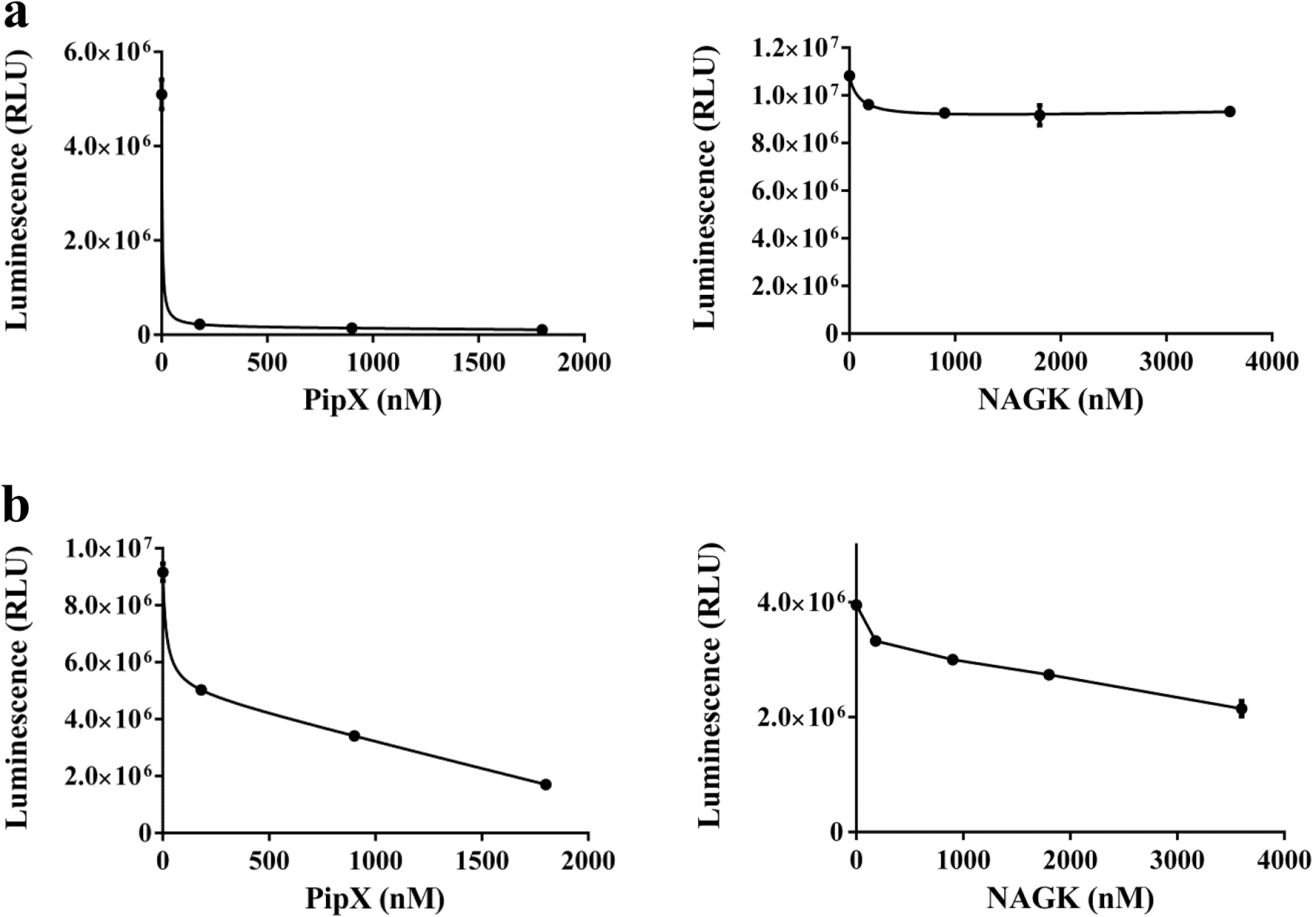
Competition between PII interaction partners to bind to PII in the presence of 2 mM effector molecules: (**a**, left panel) Competition between NAGK-SmBiT and PipX for PII-LgBiT binding in the presence of 2 mM ADP: The interaction between 10 pM PII-LgBiT (trimer) and 180 nM NAGK-SmBiT (monomer) was competed with increasing concentrations of PipX (180, 900 and 1800 nM) monomer. (a, right panel) Competition between NAGK and PipX-SmBiT for PII-LgBiT binding in the presence of 2 mM ADP: The interaction between 10 pM PII-LgBiT trimer and 10 nM PipX–SmBiT monomer was competed with the addition of increasing concentrations of NAGK (180, 900, 1800 and 3600 nM) monomer. (**b**, left panel) Competition between NAGK-SmBiT and PipX for PII-LgBiT binding in the presence of 2 mM ATP: The interaction between 10 pM PII-LgBiT (trimer) and 180 nM NAGK-SmBiT (monomer) was competed with increasing concentrations of PipX (180, 900 and 1800 nM) monomer. (**b**, right panel) Competition between NAGK and PipX-SmBiT for PII-LgBiT binding in the presence of 2 mM ATP: The interaction between 10 pM PII-LgBiT trimer and 50 nM PipX–SmBiT monomer was competed with the addition of increasing concentrations of NAGK (180, 900, 1800 and 3600 nM) monomer. Graphs represent mean ± SD of two independent experiments.

Another possible application of the NanoBiT system was to reveal competition between PipX and NAGK for PII binding. In a first experiment, PipX was titrated to a constant concentration of PII-LgBiT and NAGK-SmBiT (10 pM trimer and 180 nM monomer, respectively) in the presence of 2 mM ADP. As expected from higher affinity of PII to PipX than to NAGK in presence of ADP (compare Tab.1), the addition of PipX completely abolished luminescence of the PII-LgBiT-NAGK-SmBiT complex (Fig. 5a left panel). Vice versa, addition of increasing NAGK amounts to a constant concentration of PII-LgBiT and PipX-SmBiT in presence of ADP showed no effect on the luminescence signal, clearly indicating that in presence of ADP, PII exclusively binds to PipX, when both partners are present (Fig. 5a, right panel). A more complicated situation was observed when the same set of experiments was performed in presence of ATP. Titrating PipX to the PII-LgBiT and NAGK-SmBiT sensor pair resulted in a gradual decrease in luminescence. Although the affinity of PII to PipX is lower than towards NAGK in presence of ATP, the addition of a 1:1 stoichiometric amount of PipX to NAGK reduced the luminescence signal to about 50% and higher amounts of PipX decreased the signal further (Fig. 5b, left panel). Conversely, the PII-LgBiT and PipX-SmBiT sensor pair was quite resistant towards the addition of NAGK. Even a large excess of NAGK over PipX reduced the signal from the PII-LgBiT and PipX-SmBiT sensor pair only partially, although at these high NAGK concentrations, PII is expected to preferentially interact with NAGK (Fig. 5b, right panel). A possible explanation could be a weak interaction of PipX-SmBiT protein to PII-LgBiT-NAGK complexes, resulting in luminescence signals, due to the high sensitivity of the reporter system.

### Affinity of NAGK to PII variants in the presence of effector molecules

**Figure 6:**
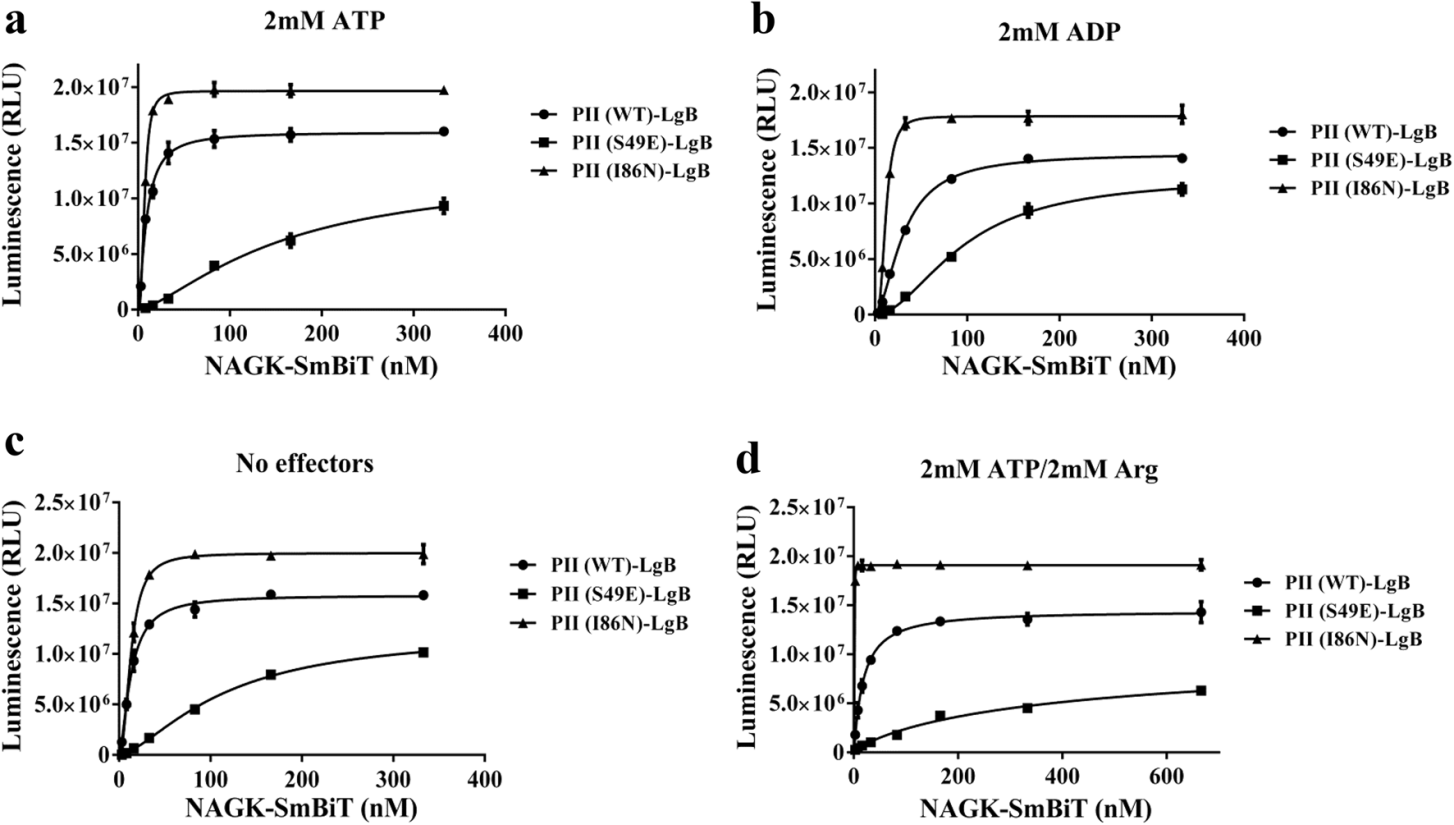
Determination of complex formation between PII-LgBiT variants (WT, S49E, I86N) and NAGK-SmBiT measured by NanoBiT-sensor. (**a**) NAGK-SmBiT titration in the range of 3–333 nM with 10 pM PII-LgBiT variants (WT, S49E, I86N) in the presence of 2 mM ATP. (**b**) NAGK-SmBiT titration in the range of 3–333 nM with 10 pM PII-LgBiT variants (WT, S49E, I86N) in the presence of 2 mM ADP. (**c**) NAGK-SmBiT titration in the range of 3–333 nM with 10 pM PII-LgBiT variants (WT, S49E, I86N) without effector molecules. (**d**) NAGK-SmBiT titration in the range of 3–666 nM with 10 pM PII-LgBiT variants (WT, S49E, I86N) in the presence of 2 mM ATP and 2 mM Arginine. Graphs represent mean ± SD of three independent experiments.

The association kinetics of NAGK-SmBiT was analysed with two mutant PII-LgBiT variants: The T-loop variant PII-S49E was shown previously to be strongly impaired in NAGK interaction^6,12^, whereas by contrast, the PII-I86N variant was identified as a hyperactive NAGK binder^20^. As shown in Fig. 6a-c, the NanoBiT assay was able to reveal low residual binding of the phosphomimetic variant PII-S49E to NAGK in the presence of ATP, ADP or without effector molecules. Under any of these conditions, complex formation could be detected, but the affinity of the phosphomimetic variant to NAGK was about 10–20 fold lower than that of wild-type PII. By SPR, NAGK interaction with the PII-S49E was undetectable and could so-far only be observed by FRET analysis^12^. The respective dissociation constants are shown in Table 1. The PII-I86N-LgBiT has a single amino acid replacement, IIe86 to Asp86, which leads to constitutive high activation of NAGK. In agreement, the NanoBiT analysis revealed high affinity to NAGK, and importantly, the affinity remained high in the presence of ADP.

### Inhibitory effect of arginine on PII-NAGK interaction

Another effector molecule that interferes with the PII-NAGK complex is arginine. Arginine binds to allosteric sites in NAGK, one site per each subunit, which are occupied in an anti-cooperative manner, thereby inhibiting its activity^21,31^. In complex with PII, NAGK feedback-inhibition by arginine is strongly relieved, presumably because the PII complex lowers the affinity of Arg to the allosteric binding sites. To find out how arginine, conversely, affects NAGK-SmBiT-PII-LgBiT complex formation, arginine was titrated to PII-LgBiT-NAGK-SmBiT complexes and luminescence was recorded. As shown in Fig. S1, very low concentrations of arginine slightly enhanced complex formation, but with increasing concentrations of Arg (when 2 mM ATP or 1 mM ATP/1 mM ADP were present), the luminescence signal decreased gradually to a new steady state level of approx. 60% of the initial value. The same experiment in presence of 2 mM ADP revealed a slightly increasing luminescence signal at low Arg levels, which returned back to initial value at higher concentrations of Arg. In the absence of adenylnucleotides, low concentration of Arg were unable to increase complex formation and the luminescence signal (Fig. S1). These results indicate that the completely arginine-occupied NAGK has a reduced affinity for PII, whereas binding of Arg to the first allosteric site transiently increases the affinity. At a concentration of 2 mM arginine, arginine completely inhibits the activity of free NAGK and inhibits the activity of NAGK in PII-complex to about 90%. To determine the binding affinity of NAGK to PII under these assay conditions, increasing concentrations of NAGK were titrated to 10 pM of the respective PII-LgBiT variants (Fig. 6d, Quantitative data are shown in Table 1. The affinity of PII was approx twofold lower in presence of 2 mM Arg, in agreement with the reduced luminescence signal observed in the titration experiment with increasing Arg concentrations (Fig. S1). Obviously, the inhibition of enzyme activity is not caused by complex dissociation. Rather, it seems that PII can still bind to completely Arg-saturated NAGK, but the complex likely adopts a different conformation, which obstructs the catalytic activation of NAGK by PII.

Interestingly, Arg had a strong, differential effect on the affinity of NAGK towards different PII variants: Whereas on wild-type PII, it moderately reduced the affinity, an even stronger reduction was observed for the PII-S49E variant. Strikingly, the affinity towards PII-I86N variant was highly increased (Fig. 6d). This explains the strong stimulation of arginine synthesis in a *Synechocystis* strain, in which the PII wild-type protein was replaced by the PII-I86N variant, causing constitutive arginine overproduction and accumulation of cyanophycin^32^.

### Feed-forward regulation of N-acetylglutamate (NAG) on PII-NAGK complex formation

**Figure 7:**
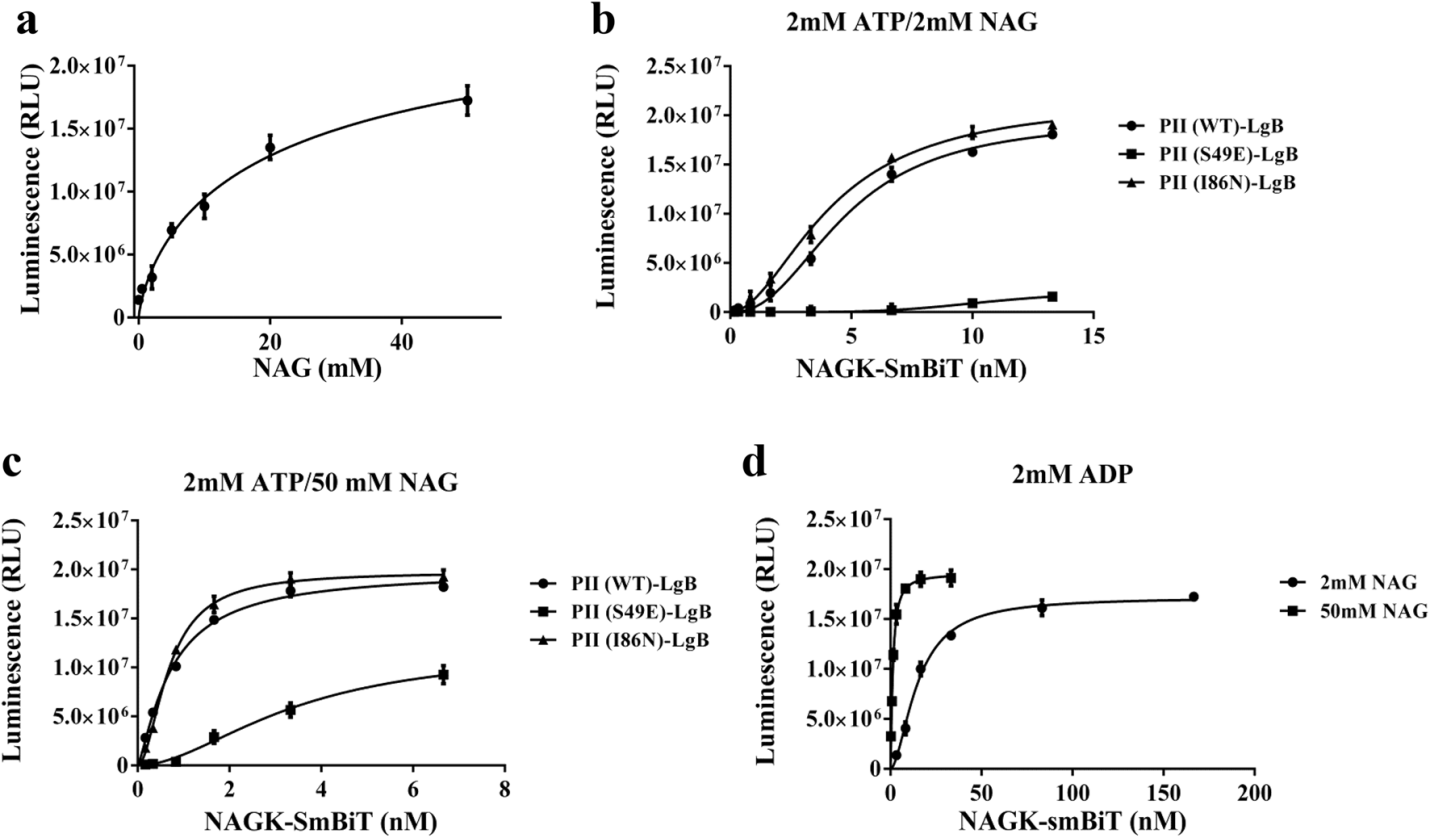
Effect of NAG on PII-NAGK interaction. (**a**): NAG titration (0–50 mM) for 10 pM PII-LgBiT and 5 nM NAGK-SmBiT in the presence of 2 mM ATP. (**b**): NAGK-SmBiT titration to 10 pM PII-LgBiT variants in the presence of 2 mM ATP/ 2 mM NAG. (**c**): NAGK-SmBiT titration to 10 pM PII-LgBiT variants in the presence of 2 mM ATP/ 50 mM NAG. (**d**): NAGK-SmBiT titration to 10 pM PII-LgBiT in the presence of 2 mM ADP with either 2 mM NAG or 50 mM NAG. Graphs represent mean ± SD of three independent experiments.

Next, we investigated how the substrate of NAGK, N-acetylglutamate (NAG), affects its interaction with PII. The influence of the substrate on the interaction of NAGK with PII was so-far never investigated, but must be taken into consideration for understanding the in vivo situation. NAG was titrated into a binding assay, whereby NAGK was used at a concentration equivalent to half Km, to enable the sensitive detection of changing affinity. As shown in Fig. 7a, increasing NAG concentrations caused a dramatic increase in PII-NAGK interaction. The binding constants were determined for a low (2 mM) and a high (50 mM) NAG concentration. 50 mM NAG is the concentration of NAG, routinely used in enzyme assays. The results are displayed in Fig. 7b,C and Table 1. NAG stimulates PII-NAGK interaction under any conditions. In the presence of ATP the affinity doubles with 2 mM NAG and increases more than tenfold with 50 mM NAG (as compared to the absence of NAG). The stimulating effect of NAG on PII-NAGK interaction is so strong that efficiently stabilizes the complex in presence of ADP (see Fig. 7d) (20-fold increase with 50 mM NAG, Table 1). The presence of Arg mitigated the strong stimulating effect of NAG on PII-NAGK affinity. In presence of 2 mM Arg, only a moderate increase by NAG on PII-NAGK interaction was detected (Fig. S2 and Table 1).

Interestingly, the S49E PII variant strongly bound to NAGK in the presence of 50 mM NAG and ATP, although this variant is unable to relieve NAGK from arginine inhibition (Fig. 7c). These results clearly demonstrate binding of PII to NAGK is not sufficient to cause a relief from Arg inhibition. The sophisticated hydrogen-bonding network organized by the –OH group of Ser49, which propagates into the catalytic centre of NAGK^6^ is required to tune the enzyme. Therefore, the weak interaction of the S49E PII variant with NAGK does not affect enzyme activity. Under in vivo conditions, the concentration of NAG is probably in the low millimolar range (as deduced from the low abundance in metabolome data, e.g.^17^. Hence, the dependence of PII-NAGK complex formation on NAG should have physiological consequences.

**Figure 8:**
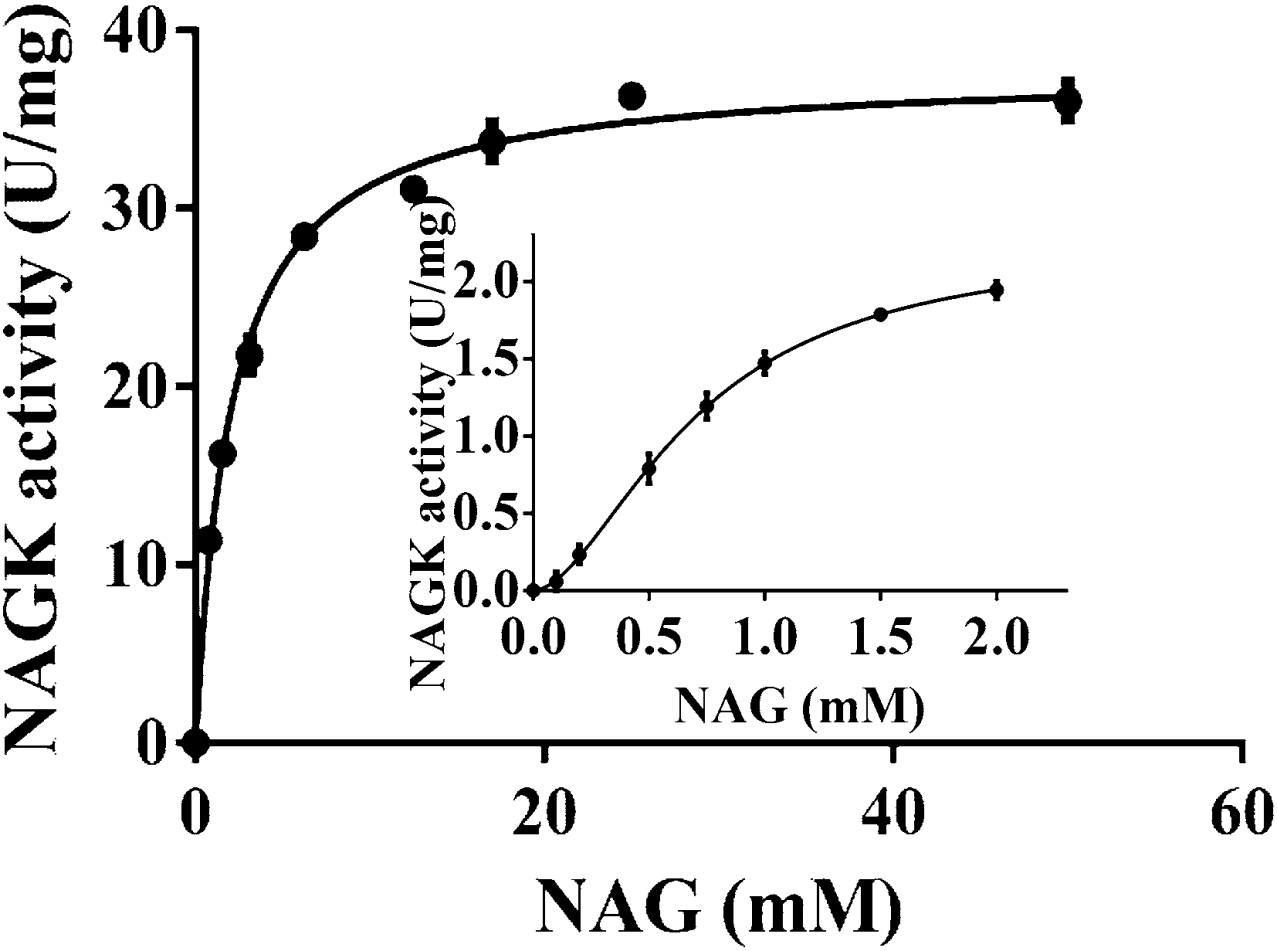
Activation of catalytic activity of NAGK by NAG in standarad NAGK assay and coupled NAGK assay. Standarad NAGK assay with NAG titration (0–50 mM) PII (1.2 μg) in the form of complex with NAGK (2 μg) in the presence of 10 mM ATP. Coupled NAGK assay with NAG titration (0–2 mM) for PII (2.4 μg) in the form of complex with NAGK (6 μg) in the presence of 1 mM ATP/1 mM ADP and 0.1 mM Arginine. Graphs represent mean ± SD of three independent experiments.

With increasing substrate concentrations, the affinity of NAGK for PII binding increases, leading to enhanced kinetic activation. Therefore, we tested whether NAGK activity in the PII complex at low NAG concentration and with 1 mM ATP/1 mM ADP and 0.1 mM Arg follows a sigmoidal curve, and in comparison, performed the standard assay in presence of 10 mM ATP (Fig. 8). The inset in Fig. 8 shows the result of the complex metabolite mixture. In fact, substrate-dependent activity follows a sigmoidal curve that could be fitted to a Hill slope of 1.76 ± 0.032. The increasing affinity of PII-NAGK with increasing concentrations of NAG results in a unique feed-forward like activation of the PII-NAGK complex by the enzyme substrate.

## Conclusions

The present analysis has established that NanoBiT is a reliable approach for the analysis of PII – receptor interactions. For the first time, we were able to accurately determine the affinity constants of PII with its target PipX and NAGK under identical experimental conditions.

The assay is robust and requires only minute amounts of protein. In contrast to surface plasmon resonance or Bio-layer interferometry analysis, the interaction occurs with proteins in solution. The only unnatural feature is the fusion of LgBiT and SmBiT fragments to the proteins of interest. Of note, the intrinsic affinity for LgBiT and SmBiT is extremely low through optimization of NanoBiT system, achieving a K_D_ value as high as 190 µM^25^. Therefore the weak association between two parts of sensor should not influence the affinity of interacting proteins which is by several orders of magnitude higher (PII-PipX and PII-NAGK complexes in our study with K_D_ values in the range of nM). The specificity of association through PII interactions is also clearly indicated by the observation of fast dissociation of the PII-PipX complex upon addition of 2-OG. (See Fig. 3c): If the LgBiT-SmBiT fragments would affect the affinity, no such rapid dissociation would be visible. The intrinsic affinity is therefore so low that it doesn’t affect the experimentally measured affinities between PII and its partner proteins. In accord, we observed almost negligible background under our experimental conditions, probably due to the low concentration of proteins used, far below the intrinsic affinities to Lg-BiT and Sm-BiT. Therefore, the emergence of luminescence can safely be interpreted as indication of complex formation.

In previous experiments on cyanobacterial PII protein interactions by Surface-Plasmon-Resonance (SPR), the K_D_ values for the PII-NAGK and PII-PipX interactions were never reported (the assays were carried out with the aim to determine the relative influence of effector molecules on PII interactions). The only K_D_ value for the PII-NAGK complex was determined previously by ultrafiltration experiments in presence of very high NAGK concentrations, so that complex formation equilibrium was almost completely on the side of the complex. From the small amount of free PII a K_D_ of approximately 73 nM was reported^6^. The K_D_ value that we here obtained by NanoBiT system (K_D_ = 12.9 nM) is almost six times lower although still in the same magnitude of order. In principle, the difference could be caused by different buffers used in these experiments. Very recently, we applied Biolayer interferometry, to measure PII interactions, whereby the interaction of PII with NAGK was tested as a proof of principle for the method (Supplementary material in Scholl et al.)^17^. Therefore, we now re-calculated the binding curves using curve fitting from association and dissociation kinetics according to Octet data analysis HT software (ForteBio) and this results in an apparent K_D_ of 11.7 ± 0.005 nM which is perfect agreement with the NanoBiT sensor data with the K_D =_ 12.9 ± 0.65 nM. This gives additional credibility to the results from the NanoBit sensor.

Due to the high sensitivity of the interaction assay, it was even possible to detect residual low-affinity interactions, such as those between the PII variant PII-S49E with NAGK, which had never been detected before with SPR or BLI analysis. From the crystal structure of the PII-NAGK complex it is known that the seryl-residue at the tip of the T-loop stabilizes the tight PII-NAGK complex and causes the enzymatic activation of NAGK by PII. A weak interaction of the PII S49 variant (PII-S49G) could, however, be detected by FRET analysis^12,13^ and was interpreted as indication of the transient encounter complex that is formed between PII and NAGK. This encounter complex was already predicted from the analysis of PII and NAGK mutants^20^. The NanoBiT study now for the first time resolved the affinity of the encounter complex, and revealed that this complex is not favoured by the state of adenyl-nucleotide binding to PII, as can be deduced from the dissociation constants of PII-S49E-NAGK in presence of different effectors. The PII-S49E variant represents a phosphomimetic variant of PII: wild-type PII is phosphorylated at S49 under nitrogen-poor conditions. The phopshomimetic variant is not able to catalytically activate NAGK, and it is reasonable to assume that likewise, also phospho-PII will not activate PII. However, the result of the NanoBiT analysis shows that the complex doesn’t dissociate completely, suggesting that upon phosphorylation of PII at the tip of the T-loop, the proteins remain loosely associated. Dephosphorylation of PII under appropriate condition would then enable an immediate re-formation of the PII-NAGK complex.

A further application of the PII-based NanoBiT toolbox is the quantification of competitive binding of different PII receptors to PII. Here, we have demonstrated adenyl-nucleotide-dependent competition between PipX and NAGK for PII binding. PII has the highest affinity for PipX in the presence of ADP (5.7 nM), whereas under these conditions, affinity for NAGK is low (30.8 nM). Therefore, not surprisingly, NAGK was not able to compete with PipX: binding of PipX-SmBiT was unaffected by the addition of NAGK. By contrast, in presence of ATP, affinity of PII to PipX is diminished (52.4 nM) concomitantly with an increased affinity for NAGK (8.8 nM). Under these conditions, addition of NAGK lowered PII-LgBiT-PipX-SmBiT interaction, reflecting successful competition. Surprisingly, however, even a large excess of NAGK was unable to abolish the signal of the PII-LgBi-PipX-SmBiT sensor pair, although PII should have been almost completely saturated with NAGK in these conditions. The remaining luminescence signal could indicate vicinity between PipX-SmBiT and PII-LgBiT bound to NAGK, but the type of interaction remains to be clarified.

Altogether, this study has demonstrated the usefulness of the NanoBiT technology to study the interactions between PII and its receptors, and in a general sense, to investigate weak and transient protein–protein interactions. Future studies will transfer the method to the analysis of the recently identified PII receptors to gain quantitative insights in PII interactions with the ultimate goal to be able to model the network of PII interactions, for which quantitative data are required.

## Methods

### Cloning and DNA purification

To generate the split NanoLuc sensor, PII-LgBiT, PipX-SmBiT and NAGK-SmBiT synthetic genes, which already contained overlapping sequences for Gibson assembly, were ordered from Integrated DNA Technologies (Biotechnology company, Leuven, Belgium) (Table S1). PII- LgBiT was cloned in pASK-IBA3 vector linearized by EcoR1 and HindIII. Two other constructs, PipX-SmBiT and NAGK-SmBiT were cloned into NdeI and BamHI sites of pTEV5 vector with N-terminal-fused His_6_-tag via Gibson cloning as described previously^33^. Plasmid DNA was amplified by transformation into *E. coli DH10β*, which was grown at 37 °C overnight on agar media containing the appropriate antibiotic for selection. Positive colonies were identified by colony PCR and then transferred to Lysogeny broth (LB) supplemented with the appropriate antibiotic and grown at 37° C overnight. Plasmid DNA was purified from cells using the Monarch Plasmid Miniprep Kit (New England Biolabs, Frankfurt a.M., Germany) according to the manufacturer's instructions. All sequences were verified using the GATC LIGHTRUN service (Eurofins Genomics, Ebersberg, Germany). Afterwards, the resulting plasmids were transformed into electrocompetent *E. coli Lemo21* cells for subsequent expression of recombinant proteins.

### Overexpression and Purification of recombinant proteins

The expression of recombinant proteins was performed by growing the cells at 37 °C under continuous shaking at 120 rpm to an OD600 of about 0.6–0.8. Depending on the plasmid the appropriated amount of the antibiotic was added to the culture. Overexpression was induced by the addition of 0.5 mM isopropyl-β-D-thiogalactopyranoside for His-tagged proteins and 0.2 μg/ml of anhydrotetracycline for Strep-tagged proteins. Induction was performed at 20 °C/120 rpm overnight. Then, the cells were harvested on the next morning by centrifugation (3.500 rpm for 15 min at 4 °C) and pellets were stored at -20˚ C until use.

The recombinant protein (PII) containing the strep_10_-tag was purified via 5 ml Strep-tactin® superflow columns. The cells were lysed in 50 ml lysis buffer containing 50 mM Tris/HCl (pH 7.4), 50 mM KCl, 5 mM MgCl_2_, 2 mM EDTA, 2 mM DTT and 0.5 mM PMSF. Ultrasonication was applied to disrupt the cells and lysate was centrifuged for 45 min at 55,000 × g at 4˚C. The supernatant was loaded on a 5 ml of Strep-Tactin Superflow column (IBA, Göttingen, Germany) that was equilibrated with the appropriate lysis buffer before use. Next, the column was washed with 100 ml of Strep washing buffer containing 100 mM Tris/HCl (pH 8.0), 150 mM NaCl, 1 mM EDTA and then it was eluted with Strep elution buffer containing 100 mM Tris/HCl (pH 8.0), 150 mM NaCl, 1 mM EDTA, 2.5 mM D-desthiobiotine. Roti-Quant (Roth, Karlsruhe, Germany) was applied to estimate the concentration of the eluted proteins in the elution fractions by following the manufacturer's instructions. The eluted protein was loaded on SDS-PAGE to check the purity and size of protein. The recombinant PII protein was dialyzed against PII dialysis buffer containing 20 mM Tris/HCl (pH 7.8), 150 mM KCl, 50% glycerol, 1 mM DTT and stored at -20˚ C until use.

The recombinant proteins (PipX and NAGK) containing His_6_-tags were purified via 1 ml Ni–NTA HisTrap columns. The purification steps were similar to those of strep tag-proteins, while the buffers and columns were different. The lysis buffer contained 50 mM Tris/HCl (pH 7.4), 50 mM KCl, 5 mM MgCl_2_, 10 mM Imidazole, 2 mM DTT and 0.2 mM PMSF. HisTrap columns were washed with 100 ml of His washing buffer 1 including 50 mM NaH_2_PO_4_ (pH 8.0), 300 mM NaCl, 20 mM Imidazole, followed by 50 ml of His washing buffer 2 with 50 mM NaH_2_PO_4_ (pH 8.0), 300 mM NaCl, 40 mM Imidazole. Elution of the bound proteins from the column with Ni–NTA was performed with an elution buffer containing 50 mM NaH_2_PO_4_ (pH 8.0), 300 mM NaCl, 250 mM Imidazole. The fractions consisting of recombinant protein were dialyzed in the respective dialysis buffer containing 50 mM Tris/HCl (pH 7.8), 100 mM KCl, 5 mM MgCl_2_, 0.5 mM EDTA, 1 mM DTT and 50% glycerol. The proteins were stored at -20˚ C for further use.

### Establishment of the bioluminescence assay

The luminescence assay for both types of measurement-time point and time course-was performed in a buffer containing 50 mM Tris/HCl (pH 7.6), 100 mM KCl, 10% glycerol, 2 mM MgCl_2_ and 0.1% BSA. Next, the sensor proteins were added to the final concentration of 10 pM (trimer) for PII-LgBiT and a range of concentrations (0–800 nM) for PipX-SmBiT. The respective concentrations of effector metabolites (ATP, ADP, and ATP/2-OG) were included to the final volume (500 µl) in the test tubes. The mixture was incubated for 15 min at 30˚ C. Afterwards, 0.5 µl of Nano-Glo® Luciferase substrate (Promega, Walldorf, Germany) per 500 µl of total volume was added to each reaction tube. The solution was incubated for 5 min at room temperature to allow the reagent and proteins to adapt to the buffer. Subsequently the luminescence was quantified in a luminometer (Sirus Luminometer, Berthold Detection System, Germany) for 10 s with 10 s delay. The recorded luminescence is reported as relative light units (RLU). For background value, every protein was measured separately. Then, the recorded signal was subtracted from the luminescence of the forming complex.

To investigate the subsequent effect of metabolites in time-course experiments for PII-LgBiT and PipX-SmBiT, 10 pM PII was added to 10 nM PipX-SmBiT in the above mentioned buffer. Then, 0.5 µl of Nano-Glo® Luciferase substrate was added to the reaction tube. After 5 min incubation, the luminescence was measured via FB12 Sirius software with 20 s intervals in the Sirus luminometer for some minutes. To monitor the effect of metabolites on complex formation, the appropriate amounts were added to the reaction tube and the measurement was continued for several minutes until a maximum binding was reached.

In the case of luminescence measurement (time point) for PII-LgBiT variants and NAGK-SmBiT, the respective concentration of proteins and metabolites were incubated for 40 min at 30˚ C. The rest of the procedure was the same as the one described above.

### Competition assay based on NanoBiT sensor

Competition assay between PipX and NAGK-SmBiT to bind to PII-LgBiT in the presence of 2 mM effector molecules, was performed with incubation of 10 pM PII-LgBiT trimer and different concentration of PipX (180, 900 and 1800 nM) monomer for 15 min at 30˚ C. Then a constant concentration of NAGK-SmBiT (180 nM) monomer was added to the mixure and incubation was continued for other 30 min at 30˚ C. Later, 0.5 µl of Nano-Glo® Luciferase substrate (Promega, Walldorf, Germany) per 500 µl of total volume was added to each reaction tube. After 5 min of incubation, luminescence was quantified in a luminometer (Sirus Luminometer, Berthold Detection System, Germany) for 10 s with 10 s delay.

Competition assay between NAGK and PipX-SmBiT to bind to PII-LgBiT was performed with incubation of 10 pM PII-LgBiT trimer and different concentration of NAGK (180, 900, 1800 and 3600 nM) monomer for 30 min at 30˚ C. Then a constant concentration of PipX-SmBiT (10 nM) monomer was added to the mixture and incubation was continued for other 15 min at 30˚ C. The final steps were performed as described above.

### AGPR coupled NAGK activity assay

To study the effect of NAG on the activity of the P_II_-NAGK complex, the specific activity of NAGK from *Synechocystis sp.* PCC 6803 was assayed by coupling NAGK-dependent NAG phosphorylation to an auxiliary enzyme (AGPR) from *E. coli*. AGPR reduced the NAG-phosphate by using NADPH as reductant and the change was recorded at 340 nm as described^34,35^. The reaction buffer consisted of 50 mM imidazole (pH 7.5), 50 mM KCl, 20 mM MgCl_2_, 0.2 mM NADPH, 0.5 mM DTT, 1 mM ATP, 1 mM ADP and 0.1 mM arginine. Each reaction contained 10 μg AGPR, 2.4 μg PII and 6 μg NAGK with different concentration of NAG (0–2 mM). The reaction was started by the addition of NAGK. Thereby, oxidation of one molecule of NADPH was recorded over a period of 10 min with a spectrophotometer (SPECORD 200, Analytik Jena). The reaction velocity was calculated with molar absorption of NADPH of Ʃ_340_ = 6178 L mol^-1^ cm^-1^ from the slope of the change of absorbance per time.

### Standard NAGK activity assay

To assess the activity of NAGK, the ADP production was coupled to the NADH oxidation using the auxiliary enzymes pyruvate kinase and lactate dehydrogenase according to previously described^35,36^. The activity of NAGK was expressed in µmol/min/mg.

### Statistical analysis

The results were performed three times to confirm reproducibility. They were expressed as the mean ± SEM of the indicated experiment. The FB12 Sirius software was used to record the luminescence in time-course experiments. Then, statistical analysis was performed by applying GraphPad Prism 6 with fitting curve “one site-specific bind with Hill slope”.

## Supplementary Information

Supplementary Information.
