# Supplementary material for: Split NanoLuc technology allows quantitation of interactions between PII protein and its receptors with unprecedented sensitivity and reveals transient interactions
Source: Sci Rep. 2021 Jun 15;11:12535. doi: 10.1038/s41598-021-91856-2 (PMC8206089; doi:10.1038/s41598-021-91856-2)
Supplement: Supplementary file 1 — Supplementary Information. [file 41598_2021_91856_MOESM1_ESM.pdf]

Split NanoLuc technology allows quantitation of interactions between PII protein and its receptors with unprecedented sensitivity and reveals transient interactions

Rokhsareh Rozbeh[1] and Karl Forchhammer[1]

[1]Interfaculty Institute of Microbiology and Infection Medicine, University of Tübingen, Auf der Morgenstelle 28, 72076 Tübingen, Germany

Supporting information

Linear vector (Restriction sites)	Gene	constructs
**pASK-IBA 3**<br>(EcoR1, HindIII)	**PII-ST-FL-LgBiT**	5′-ATGAAAAAAGTAGAAGCGATTATTCGCCCCTTTAAACTAGACGAAGTCAAAATTG<br>CGCTGGTTAATGCAGGGATCGTTGGTATGACGGTTTCTGAAGTTAGGGGCTTTGGCC<br>GTCAAAAAGGTCAAACAGAGCGTTACCGTGGCTCTGAATACACTGTTGAGTTTCTCCA<br>AAAACTCAAAATTGAGATTGTCGTCGACGAAGGACAGGTTGACATGGTTGTTGACAA<br>GTTGGTATCGGCGGCCCGCACCGGGGAAATCGGTGACGGTAAAATCTTCATCAGCCC<br>GGTGGATTCGGTGGTACGGATTCGGACAGGGGAAAAGGATACCGAAGCTATTAGCG<br>CTTGGAGCCACCCGCAGTTCGAAAAAAGCGGAGGCGGCGGAAGTAGCGGAGTGTTT<br>ACCCTGGAAGATTTCGTCGGCGACTGGGAGCAGACCGCCGCCTATAATCTGGACCAG<br>GTGTTGGAGCAAGGCGGCGTGTCAAGCCTGCTGCAGAATCTGGCTGTGTCCGTGACA<br>CCCATCCAGAGAATTGTGCGGAGCGGCGAGAACGCCCTGAAGATCGATATCCACGTG<br>ATCATCCCTTACGAGGGCCTGAGCGCCGATCAGATGGCCCAGATTGAAGAGGTGTTC<br>AAGGTGGTGTACCCCGTGGACGACCACCACTTCAAAGTGATCCTGCCTTACGGCACCC<br>TGGTCATCGATGGCGTGACCCCTAACATGCTGAACTACTTCGGCAGGCCCTACGAGG<br>GAATCGCCGTGTTCGACGGCAAAAAGATCACCGTGACCGGCACACTGTGGAACGGA<br>AACAAGATCATCGACGAGCGGCTGATCACCCCTGACGGCTCCATGCTGTTCAGAGTG<br>ACCATCAACAGCTAA-3′
**pTEV5**<br>(NdeI, BamHI)	**PipX-FL-SmBiT**	5′-ATGAGTAACGAAATTTACCTTAACCATCCGACCTTCGGGCTTTTGTATCAGATTTGT<br>TTCTTGGATGATAATCAGGAGATTTTTACTACCCTTTATGCCCAAAGGTTATTTTTTTT<br>GGTAAAAACGACGCCGAACAATACCCAATTTGAACCCATTACCAGGGCGGATGCCAA<br>ACTATTAATTGAAAATCGCCTGAGGTTTCTACGCCGGGCCGGGGATTTTAAGGCCTAT<br>GACGTGTTGGCAAAGTTACATAAAAACACTTTTAGATCTGCTGCCGCAGCGACCGGTT<br>CTGGATCTTCTGGCGGCGGAGGAATGGTCACCGGCTACCGGCTGTTCGAGGAAATCC<br>TGTAG-3′
**pTEV5**<br>(NdeI, BamHI)	**NAGK-FL-SmBiT**	5′-ATGAGCAGTACCCAAGATTATATCGGCGAGGAAGCCGCCACCAGAGTCAAAATCC<br>TCAGTGAAGCTTTGCCCTACATCCAACACTTTGCTGGCCGCACCGTGGTGGTCAAATA<br>TGGAGGGGCTGCCATGAAAGACAGCAACCTCAAAGACAAGGTAATCCGGGACATTG<br>TCTTTATGGCCTCCGTTGGCATTCGCCCGGTGGTAGTCCATGGCGGAGGGCCAGAAAT<br>TAACACCTGGCTAGATAAGGTGGGCATCGAACCCCAATTTAAAGATGGGTTACGGGT<br>CACCGATGCGGCCACCATGGATATTGTGGAAATGGTACTGGTGGGTAGAGTCAACAA<br>AGAATTGGTCAATTTAATTAACCAAGCCGGAGGAAAAGCCGTGGGACTTTGTGGCAA<br>AGACGGCCAACTGATGACCGCCCGCACCATGACTAATAAAGATGTTGGTTTTGTGGG<br>GGAGGTGAGCTCCGTTGATGCCCGGGTGGTGGAAACCTTGGTGAAAAGCGGTTATA<br>TTCCCGTCATTTCCAGTGTGGCGGCGGATGAATTTGGCCAAGCTCACAATATCAATGC<br>CGACACCTGTGCTGGGGAGTTGGCCGCAGCCCTAGGGGCAGAAAAACTAATTCTCCT<br>CACTGACACCAGGGGCATTTTGCGGGATTATAAAGACCCCTCCACCCTAATTCACAAG<br>TTAGATATTCAACAGGCGCGGGAATTGATTGGCTCCGGCATAGTGGCGGGGGGCAT<br>GATTCCCAAGGTTACCTGTTGTGTGCGTTCCCTAGCCCAGGGAGTAAGGGCGGCCCAT<br>ATTCTGGATGGCCGTTTGCCCCATGCTCTGTTGTTGGAGGTATTCACCGATCTGGGCA<br>TTGGTTCGATGATTGTGGCTTCGGGCTATGATCTCAGATCTGCTGCCGCAGCGACCGG<br>TTCTGGATCTTCTGGCGGCGGAGGAATGGTCACCGGCTACCGGCTGTTCGAGGAAAT<br>CCTGTAG-3′

Table S1. Plasmid constructs. The vector plasmids pASK-IBA 3 and pTEV 5 were digested with the restriction endonucleases superflow indicated in column 1, and the synthetic genes were subcloned in digested vectors via Gibson cloning.

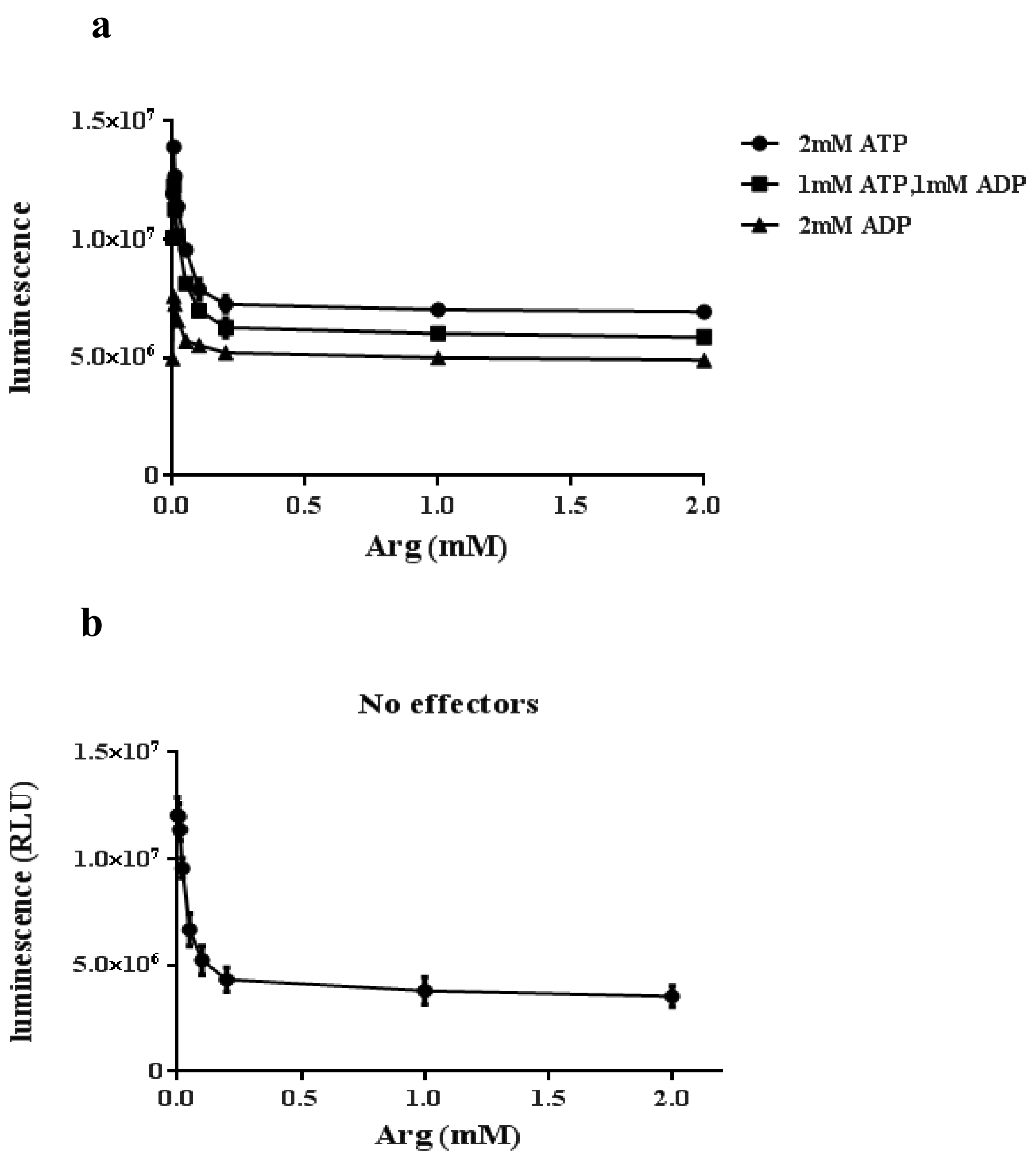

Figure S1. Effect of Arginine on complex formation between PII-LgBiT and NAGK-SmBiT. luminescence assay for 10 pM PII-LgBiT and 18 nM NAGK-SmBiT: (a) In the presence of 2mM ATP, 1mM ATP/1mM ADP and 2mM ADP and different concentrations of arginine (0, 0.005, 0.01, 0.02, 0.05, 0.1, 0.2, 1,2 mM) (b) Without effector molecules and in the presence of different concentrations of arginine (0, 0.005, 0.01, 0.02, 0.05, 0.1, 0.2, 1,2 mM). Graphs represent mean ± SD of three independent experiments.

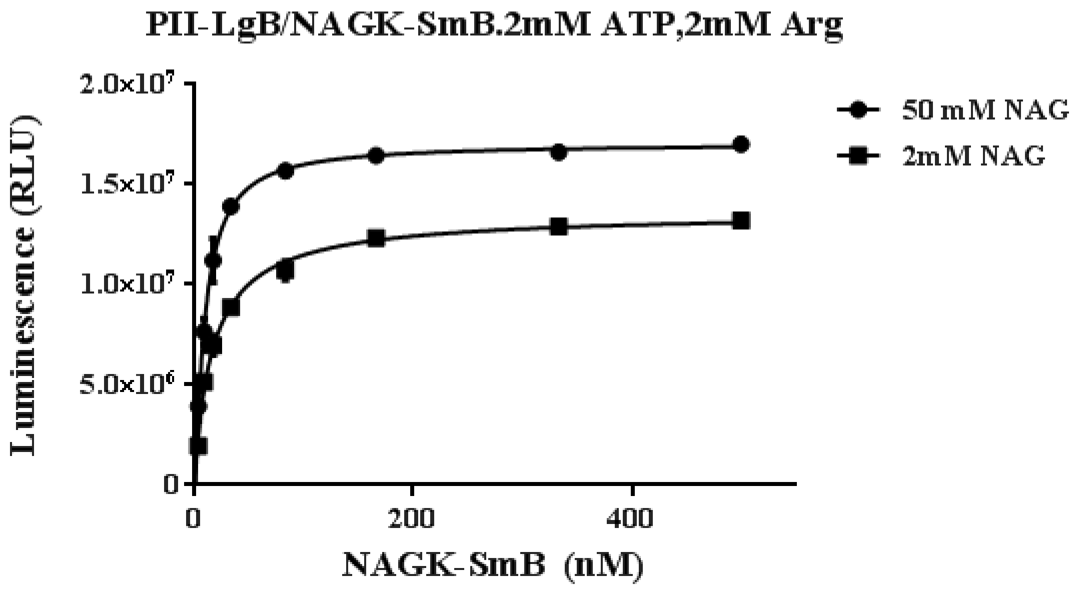

Figure S2. Determination of complex formation between PII-LgBiT and NAGK-SmBiT. In the presence of 2 mM ATP/2mM Arg and 2mM NAG or 50 mM NAG. Graphs represent mean ± SD of three independent experiments.
